# Association of health behaviour with heart rate variability: a population-based study

**DOI:** 10.1186/1471-2261-10-58

**Published:** 2010-11-25

**Authors:** Alexander Kluttig, Barbara Schumann, Cees A Swenne, Jan A Kors, Oliver Kuss, Hendrik Schmidt, Karl Werdan, Johannes Haerting, Karin H Greiser

**Affiliations:** 1 Institute of Medical Epidemiology, Biostatistics and Informatics, Martin-Luther-University Halle-Wittenberg, Halle (Saale), Germany; 2 Department of Public Health and Clinical Medicine, Centre for Global Health Research, Umeå University, Umeå, Sweden; 3 Department of Cardiology, Leiden University Medical Center, Leiden, The Netherlands; 4 Department of Medical Informatics, Erasmus Medical Center Rotterdam, Rotterdam, The Netherlands; 5 Department of Cardiology, Hospital Magdeburg, Germany; 6 Department of Medicine III, Martin-Luther-University Halle-Wittenberg, Halle (Saale), Germany; 7 Division of Cancer Epidemiology, German Cancer Research Centre, Heidelberg, Germany

## Abstract

**Background:**

Reduced heart rate variability (HRV), a non-invasive marker of autonomic dysfunction, and an unhealthy lifestyle are associated with an increased morbidity and mortality of cardiovascular diseases (CVD). The autonomic dysfunction is a potential mediator of the association of behavioural risk factors with adverse health outcomes. We studied the association of HRV with behavioural risk factors in an elderly population.

**Methods:**

This analysis was based on the cross-sectional data of 1671 participants (age range, 45-83 years) of the prospective, population-based Cardiovascular Disease, Living and Ageing in Halle (CARLA) Study. Physical activity, smoking habits, alcohol consumption and dietary patterns were assessed in standardized interviews. Time and frequency domain measures of HRV were computed from 5-min segments of highly standardized 20-min electrocardiograms. Their association with behavioural risk factors was determined by linear and non-parametric regression modelling.

**Results:**

There were only weak and inconsistent associations of higher physical activity, moderate consumption of alcohol, and non-smoking with higher time and frequency domain HRV in both sexes, and no association with dietary pattern. Results changed only marginally by excluding subjects with CVD, diabetes mellitus and use of cardioactive medication.

**Conclusion:**

We hypothesized that HRV is associated with behavioural factors and therefore might be a mediator of the effect of behavioural risk factors on CVD, but this hypothesis was not confirmed by our results. These findings support the interpretation that there may be no true causal association of behavioural factors with HRV.

## Background

Epidemiological studies provide evidence that a healthy lifestyle (e.g., physical activity, not smoking, healthy diet, and moderate consumption of alcohol) reduces morbidity and mortality due to cardiovascular causes [1]. In addition, reduced heart rate variability (HRV), a marker of autonomic dysfunction, has been shown to be associated with an increased risk of incident myocardial infarction, cardiovascular mortality, and death from other causes in general populations [2-4], and to be associated with a poor prognosis of cardiovascular diseases (CVD) [5,6]. Reduced HRV has been shown to be related to risk factors for cardiovascular disease [7-13], so autonomic dysfunction could be a mediator of the association of cardiovascular risk factors with CVD. There is some evidence that reduced HRV is amenable to intervention that may improve future health outcomes [14,15].

Several studies investigated the association of physical activity and HRV, but results are inconsistent and difficult to compare due to differences in study populations, differences in the assessment of physical activity or in training regimes, differences in recording conditions of the electrocardiograms (ECG) and due to methodological problems inherent in the analysis of HRV [9,11,15-21]. Furthermore, only few studies have analyzed the association of HRV with other behavioural risk factors such as smoking [10,11,22-24], alcohol consumption [10,11,23,25,26], and diet [10,25,27].

The Cardiovascular Disease, Living and Ageing in Halle (CARLA) study is a large population-based study with a comprehensive and highly standardized HRV measurement protocol. The aim of the present analyses was to assess the association of behavioural risk factors with HRV as the first step of the potential pathway to CVD. We hypothesized that physical activity and a favourable dietary pattern are directly associated with HRV, smoking is associated with reduced HRV, and alcohol consumption shows a nonlinear J-shaped association with HRV.

## Methods

The present analyses are based on data from the baseline examination of the prospective, population-based CARLA study. Details of the study have been described elsewhere [28,29]. In brief, the CARLA study is a prospective cohort study of a representative sample of the inhabitants of the city of Halle (Eastern Germany) comprising 1779 (812 females, 967 males) participants aged 45-83 years at baseline. The baseline examination took place between December 2002 and January 2006.

The study protocol was approved by the Ethics Committee of the Martin Luther University Halle-Wittenberg (Halle, Germany) and conforms to the tenets of the Declaration of Helsinki. All participants were informed about the study and written consent was obtained from them.

The medical examination involved recording of: blood pressure taken while seated; heart rate (HR) while seated and lying down; circumference of the waist and hip; weight and height; a 20-min 12-lead resting ECG (CardioControl Working Station, Welch Allyn, Delft, the Netherlands); and an echocardiogram. A venous blood sample was also taken.

A standardized, computer-assisted interview was undertaken to collect information on: socio-demographic and socio-economic variables; behavioural, biomedical and psychosocial factors; medical history; and use of medication within the preceding 7 days.

Physical activity was recorded using the Baecke questionnaire, describing the typically physical activity during the previous 12 months [30]. Being physically active at sport was defined as giving an affirmative answer to the question "Do you play sports?". A sport index was calculated based on the intensity, frequency and duration of sports activity [30]. Furthermore, physical activity during leisure time (excluding sport) was summarized in the leisure-time index. The total physical activity index was calculated as the sum of the sports and leisure-time index. Moreover, for the subset of participants who were characterized as being physically active in sports, we calculated the energy expenditure during sports activities in metabolic equivalents (MET) [31]. Time spent per week on each sporting activity was multiplied by the MET value of the activity to give MET-hours per week.

Information on smoking habits involved questions on: past and current smoking status; duration of smoking; and on the quantity of tobacco products smoked per day. We used two continuous measures of smoking for the present analyses, involving the number of: (i) currently smoked tobacco products per day; (ii) packyears of tobacco products ever smoked.

Self-reported usual consumption of alcohol in g/day was calculated from the answer to the questions "How much beer (in units of 0.5 l)...", "How much wine or champagne (in units of 0.2 l)...", and "How many glasses of spirits (2 cl/glass)... do you usually drink during one week?" The underlying concentration of alcohol was 4.8 volume-% for beer, 11.0 volume-% for wine or champagne, and 33.0 volume-% for spirits [32].

The mean dietary pattern during the previous 12 months was recorded by a validated food frequency questionnaire [33]. The use of selected food groups was classified according to the recommendations of the German Society of Nutrition as used in the KORA-MONICA Studies [33]. The classifications per food category were coded in an overall summary score per subject, which ranged from 0 (worst dietary pattern) to 30 (best dietary pattern). A favourable dietary pattern is characterized by a diet rich in carbohydrates (potatoes, pasta, rice and whole-grain bread), vegetables and fruits.

The 20-min ECG was recorded after a resting period (in the supine position) of ≥20 min. Throughout the ECG, subjects were asked to breathe at 15 breaths/min (0.25 Hz) - guided by a visual metronome - to standardize the influence of the respiratory rate on spectral HRV parameters.

All electrocardiograms were processed by the Modular ECG Analysis System (MEANS) [34] to obtain the location and type of the QRS complexes of the 20-min ECG. This information was used to compute standard time and frequency domain parameters of HRV for 5-min segments of the ECG according to the current guidelines for the analysis of HRV [35]. Artefacts and ectopic beats were replaced by interpolated normal sinus beats. We used the standard deviation of normal intervals (SDNN) - a time domain parameter - and the frequency domain parameters low frequency power (LF) (0.04 to <0.15 Hz), high-frequency (HF) power (0.15-0.4 Hz), and the ratio of LF to HF (LF/HF). To calculate frequency domain parameters, tachograms of RR intervals were adjusted for linear trends, tapered and zero-padded, and a Fast Fourier transformation was employed. For further analyses, HRV derived from the first 5-min segment of the 20-min ECG which fulfilled the following quality criteria was used: <10% of abnormal beats; stationarity of the tachogram; absence of atrial fibrillation, atrial flutter, artificially paced beats and other arrhythmias.

The 5-min HRV for 1671 subjects (94% of 1777 participants with a 20-min ECG) could be used for further analyses after 106 electrocardiograms had been excluded due to atrial fibrillation or flutter (n = 45), artificial pacemaker (n = 18), >10% non-sinus beats (n = 27), other abnormal rhythms (n = 4), or technical problems during processing and analysis of electrocardiograms (n = 12). For HR analyses, we used the mean value of the second and third measurement.

**Figure 1:**
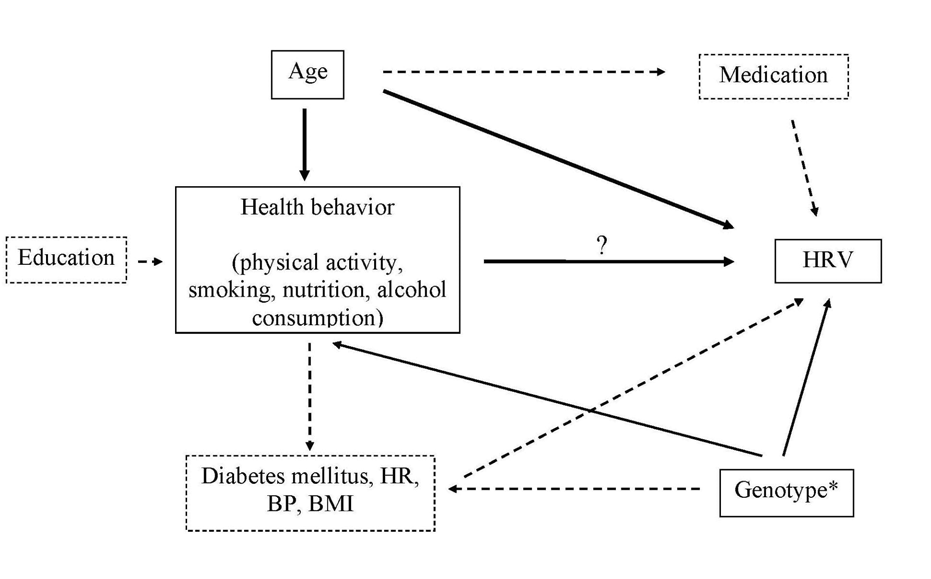
**Directed acyclic graph (DAG) as the general basis of the confounder selection for the statistical analysis. **This DAG resulted in simple age-adjusted models in the analysis of the association of healthbehaviour with HRV. BP = Blood Pressure; BMI = Body Mass Index; CVD = Cardiovascular Diseases; HR = Heart Rate; *unmeasured; solid lines: potential confounder according to DAG; dotted lines: there is no confounding according to DAG.

All HRV variables were log-transformed before analyses because of their skewed distribution. Potential confounders were selected for adjustment based on directed acyclic graphs (DAG) taking into account prior knowledge regarding their associations with health behaviours and HRV [36]. According to the resulting DAG, only age had to be adjusted for in analyses of the association of health behaviour with HRV (Figure 1). However, especially in cross-sectional studies as our study a clear temporal differentiation of cause and effect is impossible. We cannot separate the effect of health behaviour on biomedical risk factors or diseases from the effect of disease on behavioural and biomedical risk factors. For example, we cannot distinguish whether physical inactivity has caused diabetes or vice versa. We therefore performed several sensitivity analyses. For example, we additionally calculated models with adjustment for further potential confounders such as CVD, diabetes mellitus, heart rate, hypertension, body mass index, education, health behaviour, beta blockers, ACE inhibitors, diuretic, calcium channel blockers and antiarrhythmic agents.

Since categorization of originally continuous variables does not use within-category information efficiently [37], and non-linearities in the association of HRV and health behaviours were anticipated, we fitted age-adjusted generalized additive models (GAMs) [38]. GAMs can be used to check the assumption of the linearity of the relationship between HRV and health behaviours. For each smoothing effect in a model, a χ^2^-test comparing the deviance between the full model (including age and the smoothed exposure variable) and the model without the exposure variable was performed.

We calculated age-adjusted geometric means (± 95% confidence interval (CI)) of HRV parameters by categories of health-behaviour variables using linear regression models. We calculated quartiles for physical-activity indices and the dietary pattern index. For the consumption of tobacco products and alcohol, zero consumption was defined as the lowest category, and the remaining subjects were categorized in tertiles. The F-test was used to test the difference in adjusted means of HRV between categories of health-behaviour variables.

To assess the influence of prevalent disease status, we carried out sensitivity analyses of the association of HRV with health behaviour in the whole population (893 males and 778 females), as well as in a "healthy" subgroup without prevalent CVD (defined as myocardial infarction (MI), self-reported coronary artery bypass graft, self-reported percutaneous transluminal coronary angioplasty, self-reported physician-diagnosed stroke, or carotid surgery), without diabetes mellitus (defined as self-reported physician-diagnosed diabetes and/or use of anti-diabetic medication) and without HRV-relevant medication (beta-blockers, angiotensin-converting enzyme (ACE) inhibitors, anti-arrhythmic drugs) (411 males and 363 females). The association of HRV with MET-hours per week was analyzed in the subgroup of participants who played sports.

Hypothesis tests were conducted at the significance level of α = 0.01 to account for the problem of multiple testing because each independent variable was tested for its association with four HRV indices and heart rate. All analyses were undertaken using SAS 9.1 (SAS Institute, Cary, NC, USA).

## Results

The baseline characteristics of the study population are shown in Table 1. Overall, the study population showed a high burden of cardiovascular risk factors and diseases.

**Table 1:** Baseline characteristics of the CARLA study population (2002-2006)

	Women (n = 812)			Men (n = 967)		
	N	Mean (Standard deviation) or Proportion		N	Mean (Standard deviation) or Proportion	
Age (yrs)	812	63.7	(9.9)	967	64.9	(10.2)
Heart rate	812	71.5	(10.6)	966	71.34	(12.4)
LDL/HDL-Quotient	807	2.4	(0.9)	960	2.8	(1.0)
Body mass index (kg/m^2^)	812	28.5	(5.4)	967	28.5	(4.1)
Waist-to-hip ratio (WHR)	812	0.9	(0.1)	967	1.0	(0.1)
Smoking:						
Current	119	17.1%		225	27.6%	
Past	140	18.1%		496	46.9%	
Never	553	64.8%		245	25.5%	
Packyears of tobacco products	812	4.3	(9.0)	964	15.6	(16.6)
Currently smoked no. of tobacco products/day	812	1.9	(5.4)	966	3.8	(8.8)
Alcohol consumption % >20 (female)/30 (male) g/day	32	4.5%		197	22.5%	
Alcohol g/day	812	4.1	(7.1)	964	17.5	(18.67)
Sports: % active (any sports)	347	42.3%		296	31.3%	
Sport-index	808	2.4	(0.7)	962	2.4	(0.7)
Leisure-time-index	810	3.1	(0.6)	966	3.1	(0.6)
MET-hours per week^1^	347	9.6	(7.6)	296	13.1	(10.4)
Dietary pattern index	812	16.4	(3.2)	965	14.5	(3.2)
Education (years of training):						
<= 10 years	122	13.6%		36	4.2%	
11-13 years	387	47.0%		391	42.0%	
14-17 years	231	29.1%		335	32.7%	
>= 18 years	72	10.2%		205	21.1%	
Drug use:						
Betablockers	279	33.2%		307	27.6%	
ACE-Inhibitors	254	28.5%		340	30.2%	
Disease prevalence:						
Myocardial infarction (MI)^2^	17	1.9%		88	7.6%	
Stroke	27	2.8%		42	3.5%	
Cardiovascular Disease (CVD)^3^	48	5.2%		153	12.7%	
Hypertension^4^	608	71.2%		789	78.7%	
Diabetes mellitus^5^	120	13.1%		154	15.9%	

^1^MET: metabolic equivalent

^2^MI: defined as self-reported physician-diagnosed myocardial infarction and/or definite MI by physician-validated Minnesota Code of the 10-second ECG

^3^CVD: including prevalent myocardial infarction, coronary artery bypass graft, percutaneous transluminal coronary angioplasty, stroke, carotid surgery

^4^Hypertension defined as SBP >= 140 and/or DBP >= 90 mmHg, and/or use of antihypertensive medication by ATC code

^5^Diabetes defined as self-reported physician-diagnosed diabetes mellitus and/or use of anti-diabetic medication by ATC code

Table 2 and Table 3 show the age-adjusted means of HRV and HR by categories of health behaviour in the total population. There was no consistent or statistically significant association of dietary pattern, alcohol consumption and physical activity with HRV in either sex. However, males and females with a high sport index and total activity index (fourth quartile) showed a significantly lower HR compared with other quartiles. Males who never smoked (zero packyears) had a significantly lower HR, and higher SDNN, LF and LF/HF compared with smokers with any accumulated packyears. However, there was no dose-response relationship across tertiles of packyears. No statistically significant association was found with the number of currently smoked tobacco products except for HR and LF/HF in males.

**Table 2:** Age-adjusted mean heart rate variability and heart rate by categories of health behavior in the female CARLA study population

			Heart rate		SDNN		LF/HF		HF		LF	
		
Risk factor		N	Mean (95%CI)		Mean (95%CI)		Mean (95%CI)		Mean (95%CI)		Mean (95%CI)	
**Sports-index**	Q1	226	71.4	(70.1-72.7)†	27.6	(26.0-29.3)	1.0	(0.9-1.1)	162.7	(139.7-189.6)	166.3	(144.5-191.4)
	Q2	193	73.3	(71.9-74.8)	28.1	(26.3-30.0)	1.1	(1.0-1.3)	154.3	(130.7-182.1)	174.9	(150.2-203.6)
	Q3	143	71.1	(69.5-72.8)	28.1	(26.1-30.3)	1.0	(0.8-1.1)	175.1	(144.5-212.3)	172.0	(144.1-205.2)
	Q4	212	70.2	(68.8-71.6)	28.4	(26.7-30.3)	1.0	(0.9-1.1)	179.3	(153.1-210.0)	178.8	(154.6-206.7)

**Leisure time-index**	Q1	232	72.2	(70.9-73.5)	28.5	(26.8-30.2)	1.0	(0.9-1.2)	170.2	(146.2-198.1)	183.3	(159.5-210.7)
	Q2	124	71.4	(69.6-73.2)	26.1	(24.0-28.3)	1.0	(0.9-1.2)	142.1	(115.4-174.9)	142.1	(117.5-172.0)
	Q3	253	71.6	(70.3-72.9)	28.8	(27.2-30.5)	1.0	(0.9-1.1)	173.7	(150.2-200.9)	176.5	(154.5-201.7)
	Q4	167	70.5	(68.9-72.1)	28.2	(26.2-30.2)	1.0	(0.9-1.2)	179.5	(149.9-214.8)	182.4	(154.6-215.1)

**Total activity-index**	Q1	215	71.8	(70.4-73.1)†	28.0	(26.3-29.8)	1.0	(0.9-1.2)	163.1	(139.4-190.7)	169.3	(146.6-195.5)
	Q2	140	74.0	(72.3-75.7)	27.5	(25.5-29.7)	1.1	(1.0-1.3)	148.7	(122.4-180.5)	171.2	(143.2-204.7)
	Q3	194	70.7	(69.2-72.1)	27.3	(25.6-29.1)	1.0	(0.9-1.1)	162.5	(137.8-191.6)	165.8	(142.5-193.0)
	Q4	224	70.4	(69.1-71.8)	29.1	(27.4-30.9)	1.0	(0.9-1.1)	189.4	(162.5-220.9)	183.9	(159.7-211.9)

**No. of currently smoked tobacco products/day**	none	661	71.5	(70.7-72.3)	28.3	(27.3-29.3)	1.0	(0.9-1.1)	168.8	(154.2-184.8)	175.8	(161.8-191.0)
	T1	37	70.9	(67.6-74.3)	27.5	(23.7-32.0)	1.3	(1.0-1.7)	153.8	(105.0-225.4)	196.6	(138.5-279.2)
	T2	46	71.3	(68.3-74.4)	27.5	(24.0-31.5)	0.8	(0.6-1.1)	185.2	(131.2-261.4)	153.5	(111.9-210.7)
	T3	34	74.3	(70.7-77.8)	26.8	(22.8-31.4)	0.9	(0.7-1.2)	154.8	(103.6-231.4)	144.9	(100.2-209.5)

**Packyears of smoking**	none	482	72.1	(71.2-73.1)	28.3	(27.1-29.5)	1.1	(1.0-1.2)	164.4	(147.8-182.8)	176.9	(160.5-195.1)
	T1	98	70.1	(68.1-72.2)	28.3	(25.8-31.0)	1.0	(0.9-1.2)	182.8	(144.7-230.9)	188.5	(152.2-233.6)
	T2	100	70.7	(68.7-72.8)	28.0	(25.5-30.7)	1.0	(0.8-1.1)	173.2	(137.1-218.9)	168.5	(135.9-208.8)
	T3	98	71.5	(69.5-73.6)	27.3	(24.9-30.0)	0.9	(0.8-1.1)	169.4	(133.8-214.5)	151.9	(122.4-188.7)

**Alcohol (g/d)**	none	428	71.4	(70.4-72.4)	28.0	(26.8-29.3)	1.0	(0.9-1.1)	168.3	(150.4-188.4)	172.8	(155.9-191.6)
	T1	91	73.9	(71.7-76.0)	27.6	(25.1-30.4)	1.0	(0.8-1.2)	168.0	(131.8-214.2)	169.0	(135.3-211.2)
	T2	145	70.7	(69.0-72.4)	28.0	(25.9-30.2)	1.0	(0.9-1.2)	166.8	(137.5-202.3)	173.0	(144.9-206.5)
	T3	114	71.9	(70.0-73.8)	29.1	(26.7-31.7)	1.1	(0.9-1.2)	170.7	(137.4-212.2)	183.1	(149.9-223.5)

**Dietary pattern index**	Q1	215	71.4	(70.0-72.8)	30.0	(28.2-32.0)	1.1	(1.0-1.3)	188.2	(160.7-220.4)	210.9	(182.5-243.7)
	Q2	191	72.1	(70.6-73.6)	26.9	(25.1-28.7)	1.1	(0.9-1.2)	144.8	(122.5-171.1)	155.2	(133.2-180.8)
	Q3	176	71.1	(69.6-72.7)	27.6	(25.7-29.5)	1.0	(0.8-1.1)	165.8	(139.4-197.3)	159.0	(135.6-186.4)
	Q4	196	71.8	(70.4-73.3)	27.8	(26.0-29.7)	1.0	(0.9-1.1)	174.9	(148.3-206.3)	170.3	(146.4-198.1)

Q1 = lowest quartile of health behavior, Q4 = highest quartile of health behavior; T1 = lowest tertile of health behavior (excluding non-consumer), T3 = highest tertile of health behavior (excluding non-consumer); † p < 0.01, ‡ p < 0.001 (F-test for difference between respective categories of health behavior)

**Table 3:** Age-adjusted mean heart rate variability and heart rate by categories of health behavior in the male CARLA study population

			Heart rate			SDNN			LF/HF			HF		LF		
		
Risk factor		N	Mean (95%CI)			Mean (95%CI)			Mean (95%CI)			Mean (95%CI)		Mean (95%CI)		
**Sports-index**	Q1	243	72.6	(71.1-74.1)	†	25.8	(24.2-27.6)		1.6	(1.4-1.8)		102.8	(87.1-121.2)	162.8	(140.7-188.3)	
	Q2	255	72.0	(70.5-73.5)		24.5	(23.0-26.1)		1.6	(1.4-1.8)		100.2	(85.3-117.8)	158.5	(137.5-182.8)	
	Q3	177	71.9	(70.1-73.7)		27.6	(25.6-29.8)		1.6	(1.4-1.8)		119.5	(98.4-145.1)	193.1	(162.7-229.1)	
	Q4	215	69.3	(67.7-70.9)		27.2	(25.4-29.2)		1.6	(1.4-1.8)		121.3	(101.7-144.6)	198.0	(169.5-231.2)	

**Leisure time-index**	Q1	181	71.5	(69.8-73.3)		25.6	(23.8-27.7)		1.5	(1.3-1.7)		113.6	(93.7-137.6)	166.1	(140.2-196.7)	
	Q2	234	73.1	(71.6-74.7)		26.0	(24.3-27.8)		1.7	(1.5-1.9)		104.2	(88.0-123.4)	173.1	(149.1-200.9)	
	Q3	269	70.1	(68.6-71.5)		26.1	(24.5-27.8)		1.5	(1.4-1.7)		111.6	(95.4-130.6)	172.8	(150.4-198.6)	
	Q4	208	71.3	(69.7-72.9)		26.6	(24.8-28.6)		1.7	(1.5-1.9)		108.8	(91.0-130.2)	188.1	(160.6-220.4)	

**Total activity-index**	Q1	182	73.6	(71.8-75.3)	†	24.9	(23.1-26.8)		1.5	(1.3-1.7)		102.4	(84.6-124.0)	155.2	(131.1-183.6)	
	Q2	236	71.9	(70.4-73.5)		25.1	(23.5-26.8)		1.6	(1.5-1.8)		98.7	(83.5-116.8)	160.6	(138.5-186.3)	
	Q3	274	71.0	(69.5-72.4)		27.3	(25.7-29.0)		1.6	(1.4-1.7)		120.5	(103.2-140.9)	188.5	(164.4-216.3)	
	Q4	198	69.7	(68.0-71.3)		27.0	(25.1-29.0)		1.7	(1.5-1.9)		115.0	(95.8-138.2)	196.3	(167.0-230.8)	

**No. of currently smoked tobacco products/day**	none	677	70.5	(69.6-71.4)	‡	26.3	(25.3-27.3)		1.6	(1.5-1.8)	†	108.8	(98.4-120.3)	178.7	(163.6-195.3)	
	T1	77	73.3	(70.6-76.0)		25.9	(23.1-29.1)		1.8	(1.5-2.1)		100.0	(74.5-134.2)	177.0	(136.5-229.5)	
	T2	89	73.9	(71.3-76.4)		24.9	(22.3-27.8)		1.5	(1.2-1.8)		106.7	(80.6-141.2)	161.2	(125.8-206.4)	
	T3	49	77.8	(74.3-81.2)		26.5	(22.8-30.8)		1.0	(0.8-1.3)		140.9	(96.6-205.5)	148.0	(106.1-206.6)	

**Packyears of smoking**	none	191	70.0	(68.3-71.7)	†	29.2	(27.1-31.4)	†	1.7	(1.5-2.0)	†	134.3	(111.5-161.7)	232.6	(197.6-274.0)	‡
	T1	237	70.9	(69.3-72.4)		26.0	(24.3-27.8)		1.7	(1.5-1.9)		104.5	(88.4-123.5)	176.2	(152.1-204.1)	
	T2	229	70.8	(69.3-72.4)		24.7	(23.1-26.4)		1.7	(1.5-1.9)		91.5	(77.2-108.5)	158.5	(136.5-184.0)	
	T3	233	73.8	(72.3-75.3)		25.3	(23.7-27.0)		1.3	(1.2-1.5)		115.9	(97.9-137.2)	152.7	(131.7-177.1)	

**Alcohol (g/d)**	none	190	71.4	(69.7-73.1)		25.7	(23.9-27.6)		1.4	(1.3-1.6)		109.9	(91.2-132.5)	159.1	(135.0-187.6)	
	T1	237	71.5	(70.0-73.1)		25.6	(23.9-27.3)		1.7	(1.5-1.9)		101.0	(85.4-119.5)	170.6	(147.2-197.9	
	T2	226	69.9	(68.3-71.5)		27.7	(25.9-29.6)		1.6	(1.4-1.8)		129.6	(109.2-153.8)	204.7	(176.0-238.0)	
	T3	237	72.7	(71.2-74.3)		25.6	(24.0-27.4)		1.7	(1.5-1.9)		101.0	(85.3-119.5)	168.6	(145.3-195.7)	

**Dietary pattern index**	Q1	252	72.2	(70.7-73.8)		25.1	(23.5-26.8)		1.5	(1.4-1.7)		105.6	(89.4-124.7)	164.0	(141.6-189.9)	
	Q2	215	71.6	(69.9-73.2)		27.6	(25.8-29.6)		1.7	(1.6-2.0)		116.4	(97.6-138.8)	204.2	(174.8-238.4)	
	Q3	194	72.1	(70.4-73.8)		25.6	(23.8-27.5)		1.7	(1.5-1.9)		100.1	(83.1-120.5)	169.9	(144.2-200.1)	
	Q4	230	70.0	(68.4-71.5)		26.3	(24.6-28.2)		1.4	(1.3-1.6)		115.1	(96.9-136.8)	166.5	(143.0-193.9)	

Q1 = lowest quartile of health behavior, Q4 = highest quartile of health behavior; T1 = lowest tertile of health behavior (excluding non-consumer), T3 = highest tertile of health behavior (excluding non-consumer); † p < 0.01, ‡ p < 0.001 (F-test for difference between respective categories of health behavior)

**Figure 2:**
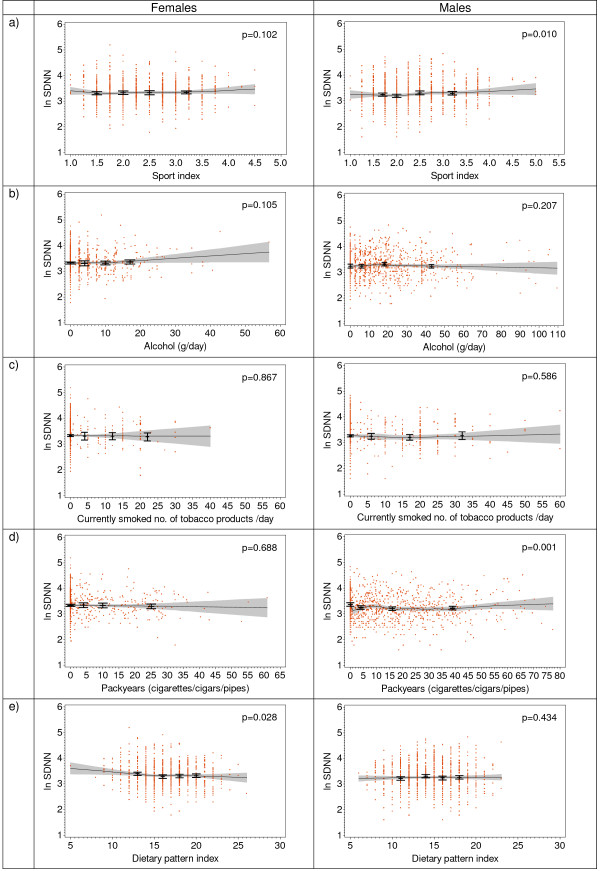
**SDNN (log transformed) in the total population by sex and health behaviour: a) by sport activity index, b) by alcohol consumption, c) by currently smoked tobacco products, d) by ever smoked tobacco packyears, e) by dietary pattern index**. Bars represent age-adjusted SDNN means (± 95% CI) by categories of health behaviour variables, lines represent loess curves (± pointwise 95% CI), p-values were calculated by χ^2^-test comparing the deviance between the age-adjusted exposure model and the model without the exposure variable.

GAM analyses confirmed the association of SDNN with packyears of smoking in both sexes (Figure 2) and the association of HR with currently smoked tobacco products and packyears in males. Furthermore, there was a weak (but statistically significant) positive association of sport index with HR, SDNN and with LF power in males. No further statistically significant associations between health behaviour indices and HRV parameters were observed.

**Figure 3:**
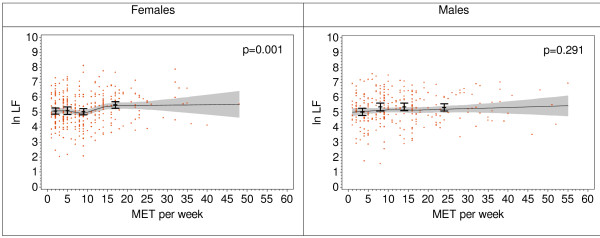
**LF (log transformed) by sex and health behaviour in participants who play sports (275 males and 332 females)**. Bars represent age-adjusted LF means (± 95% CI) by categories of MET-hours per week, lines represent loess curves (± pointwise 95% CI), p-values were calculated by χ^2^-test comparing the deviance between the age-adjusted model and the model without the MET-hours per week variable.

Analyses of the association of HRV with MET-hours per week in participants performing sport showed no significant association of SDNN, HF, and LF/HF with MET-hours per week. However, a weak (but statistically significant) association of MET-hours per week with LF was observed in females (Figure 3).

To exclude the influence of prevalent diseases and potential confounders on the association of behavioural variables and HRV, we calculated age-adjusted models in healthy subjects, and we calculated multivariate adjusted models in the whole group (adjustment for age and additionally for CVD, diabetes mellitus, HR, education, hypertension, body mass index, beta blockers, ACE inhibitors, diuretics, calcium-channel blockers and anti-arrhythmic agents). However, there was no relevant change in the association of behavioural variables with HRV (data not shown).

## Discussion

We aimed to provide population-based data on the association of HRV with behavioural risk factors in an elderly general population. We found only weak and inconsistent associations of HRV with physical activity, smoking, alcohol consumption and dietary pattern. To our knowledge, the CARLA study is the largest population-based study with such a comprehensive protocol to increase reliability and standardization of HRV measurements. Our results thus add to the literature by providing more reliable estimates of true HRV values. This enables study of the associations between behavioural factors and HRV with less bias due to measurement error and variability in examination conditions.

A limitation of our analyses was its cross-sectional nature, which hampers evaluation of causal inferences. HRV was assessed simultaneously with prevalent disease and health behaviour, so we cannot exclude the possibility that prevalent disease may have affected the levels of health behaviour and HRV. We addressed this limitation by conducting subgroup analyses in healthy subjects and additionally by calculating multivariate adjusted models in the whole group. However, we did not find relevant changes in the lack of associations.

Measurement of behavioural factors by questionnaire is susceptible to misclassification [39,40]. It cannot be ruled out that the lack of association of behavioural risk factors with HRV in the present study occurred due to measurement error in self-reported behaviours (particularly with respect to physical activity and dietary pattern). However, the used questionnaires have been validated [30,33,41] and are frequently used.

In general, comparison of the present HRV results with the literature must be done cautiously because of methodological differences. In particular, the wide range of recording conditions of applied ECG as well as the lengths and pre-processing steps used before spectral analyses may result in considerable differences in the HRV parameters themselves between studies. Furthermore, a limitation generally true for studies of HRV is that spectral parameters are sensitive to several physiological and environmental influences as well as to the pre-processing protocol [42]. Moreover, within- and between-subject reliability of HRV measurements can be poor [43]. However, in the present study, environmental conditions during ECG recording were controlled and standardized to improve reliability.

The literature is inconsistent with respect to the effect of physical activity on HRV. Physical activity causes a resting bradycardia that is thought to be partly due to the higher vagal tone of HR, thus increasing in particular HF and total HRV power. Some studies showed a positive association of physical activity with some or all HRV measures examined [7,9,15,16,19,21,44], but others did not [17,18]. A possible explanation for these conflicting results may be that, in studies showing no association of physical activity with HRV, the frequency, duration and intensity of physical activity or physical training may have been insufficient to increase vagal regulation of HR. This interpretation is supported by studies which showed that a short period of intense aerobic training as well as long-term (but low-intensity) endurance training did not cause any change in resting HRV [18,45]. In contrast, a 6-month randomized trial of moderate-intensity training (8 kcal/kg per week) revealed increased HRV in post-menopausal sedentary women [44]. A further explanation for conflicting results might be that the effect of physical activity on HRV is modified by age. Older subjects showed smaller effects than younger ones, which might be due to a reduced "trainability" of the heart [19]. However, our results showed no modification of effects by age (data not shown).

A methodological explanation for discrepant results regarding physical activity and HRV might be that positive associations tended to be derived primarily from long-term ECG recordings. This could be due to the variability of current physical activity during long-term ECG recording, which influences HRV measurements particularly low-frequency HRV [46].

Numerous studies with different recording lengths of ECG and different populations showed a negative association of smoking with various measures of HRV [3,10-12,22-24], although significant effects were not always observed for all HRV measures determined [8,11,12,24] or for both sexes [20], or were not confirmed in multivariable analyses [8]. Stolarz et al. found conflicting results for different populations [9], and no association was observed in the Rotterdam Study [4]. In the present study, we also did not find a clear pattern of a smoking effect on HRV, and the observed effects of smoking were mostly small. We assumed that current HRV is mostly affected by current smoking, but we did not find an association with the currently smoked number of tobacco products. In contrast, we found significantly higher HRV in males who never smoked compared with smokers with any accumulated packyears. This argues for a long-term effect of smoking on autonomic function.

Findings regarding alcohol consumption and HRV are inconsistent. Some authors reported a positive association in women [22,26], but others found a negative association [10,11] or no association [4,23,25]. Our results are in accordance with the latter findings. One problem impeding real comparability of the studies is that alcohol consumption was defined differently across studies with varying cutoff points for exposure classification. Perhaps measures of patterns of alcohol consumption (e.g., binge drinking) would be more relevant for the association of HRV and CVD risk.

Only one study found an association of reduced HRV with an "unhealthy eating pattern" - defined as frequent consumption of white bread and full-fat milk (instead of low-fat milk) and little consumption of fruits - in men [10]. Several authors evaluated the effect of unsaturated fatty acids on HRV [25,27,47]. Christensen et al. found a positive association of SDNN with increased consumption of fish [25]. However, a randomized trial of the effect of the consumption of industrially produced trans-fatty acids and n-3-unsaturated fatty acids on HRV did not replicate this association [27]. In the CARLA study, we did not find an association of HRV with dietary pattern and with fish consumption.

A possible explanation of our overall results could be that HRV is primarily determined by genetic factors [48-50]. A recent study found that genetic factors accounted for a major portion of the inter-individual differences in HRV, and no single behavioural determinant appeared to have a major influence on HRV [50]. Another possibility is that HRV is not a mediator of the effect of behavioural risk factors on CVD, but becomes effective after the stage of biomedical risk factors. Thus, it could just be a marker of ill health and for prospective adverse health outcomes. HRV may therefore have been found to be more strongly related to those biomedical risk factors which have a high predictive power for future CVD events (e.g., diabetes and obesity) [10,20].

## Conclusion

We hypothesized that HRV is associated with behavioural factors and therefore might be a mediator of the effect of behavioural CVD risk factors on CVD events, but this hypothesis was not confirmed by our results. These findings support the interpretation that there may be no true causal association of behavioural factors with HRV, which is also compatible with the inconsistent literature. However, a final conclusion as to whether HRV is an intermediate on the causal path or a marker of subclinical or impending disease cannot be drawn on the basis of cross-sectional analyses. Fortunately, the ongoing follow-up investigation of the study subjects will give us the opportunity to examine the prospective associations of behavioural risk factors with HRV and incident events, and to assess correct temporal relations.

## Abbreviations

BP: Blood Pressure; CARLA: Cardiovascular Disease, Living and Ageing in Halle; CVD: Cardiovascular Disease; DAG: Directed Acyclic Graph; ECG: Electrocardiogram; GAM: Generalized Additive Model; HF: High Frequency Power; HR: Heart Rate; HRV: Heart Rate Variability; LF: Low Frequency Power; LF/HF: Ratio of Low Frequency Power to High Frequency Power; MET: Metabolic Equivalent; MI: Myocardial Infarction; SDNN: Standard Deviation of Normal Intervals; 95% CI: 95% Confidence Interval.

## Competing interests

The authors declare that they have no competing interests.

## Authors' contributions

AK: conducted the statistical analyses and drafted the manuscript. BS: helped designing major parts of the study and helped drafting the manuscript. CAS: helped designing the protocol, specifically the 20 minute HRV measurement protocol with metronome guided respiration; performed the HRV analyses and helped drafting the manuscript. JAK: performed the pre-processing of ECGs for HRV analysis and the Minnesota Coding and helped drafting the manuscript. OK: participated in the statistical analyses and helped drafting the manuscript. HS: validated ECG-based diagnoses and critically reviewed the manuscript. KW: helped designing the study, and drafting the manuscript. JH: helped designing the study, selecting the statistical procedures and drafting the manuscript. KHG: conceived of the study, designed major parts of the study and helped drafting the manuscript. All authors read and approved the final manuscript.

## Pre-publication history

The pre-publication history for this paper can be accessed here:

http://www.biomedcentral.com/1471-2261/10/58/prepub
